# Prevalence of rifampicin resistance by automated Genexpert rifampicin assay in patients with pulmonary tuberculosis in Yenagoa, Nigeria

**DOI:** 10.11604/pamj.2018.29.204.14579

**Published:** 2018-04-06

**Authors:** Peter Ogie Ikuabe, Ikenna Desmond Ebuenyi

**Affiliations:** 1 Department of Internal Medicine Niger Delta University Teaching Hospital Okolobiri, Bayelsa State, Nigeria; 2 Athena Institute, Vrije Universiteit Amsterdam, The Netherlands

**Keywords:** Tuberculosis, MDR-TB, Genexpert, rifampicin resistance

## Abstract

**Introduction:**

The diagnosis of tuberculosis and its treatment is challenging in resource – limited settings. The growth and speed of multi drug – resistant tuberculosis (MDR-TB) in high burden countries like Nigeria is a growing concern. This study is aimed at determining the prevalence of rifampicin resistance in sputum specimens of patients with pulmonary tuberculosis in Yenagoa, Nigeria.

**Methods:**

A descriptive survey of all consecutive sputum specimens of adults greater than 15 years of age that presented to the Tuberculosis Referral Hospital Laboratory were subjected to the automated Genexpert test between January and December 2016.

**Results:**

All 446 specimens were tested using the Genexpert automated system. 102 (22.9%) of the sputum specimens were positive for Mycobacterium tuberculosis, with 15 (14.7%) showing rifampicin resistance.

**Conclusion:**

There was significantly high prevalence of MDR-TB much higher than the World Health Organisation (WHO) prediction of 3.2 -5.4% for Nigeria.

Pan African Medical Journal – ISSN: 1937- 8688 (www.panafrican-med-journal.com)

Published in partnership with the African Field Epidemiology Network (AFENET). (www.afenet.net)

Pan African Medical Journal – ISSN: 1937- 8688 (www.panafrican-med-journal.com)

Published in partnership with the African Field Epidemiology Network (AFENET). (www.afenet.net)

## Introduction

Multidrug resistant TB (MDR-TB), defined by resistance to isoniazid and rifampicin (RIF), the two key first- line anti- TB drugs in short course chemotherapy [1, 2]. MDR-TB is a growing global health problem [3-6]. While MDR-TB emerges as a consequence of poor adherence to anti-TB treatment [1, 2], it can also be transmitted. The estimates based on modeling predict MDR-TB prevalence in Nigeria to range from 4. 3% (3.2-5.4%) for new cases up to 25% (19-31%) for previously treated patients [7]. The global control of tuberculosis is hampered by slow, insensitive diagnostic methods, particularly for the detection of drug- resistant forms. Early detection is essential to reduce the death rate and interrupt transmission, but the complexity and infrastructure needs of sensitive methods limit their accessibility and effect [1, 4]. The conventional methods of drug resistance testing involve growth of *Mycobactericum tuberculosis* on Liquid of solid culture medium and takes about 8 weeks [1]. Culture methods are costly and time consuming, thus limiting both utility for patients care and likelihood of timely treatment. Lately, several new commercial tests have been described that identify MDR-TB based on the genetic sequence. One of such is used in the present study which diagnoses TB and identifies drug resistance. It specifically examines samples for classic, genetic mutations that confer resistance to both isonazid and rifampicin. Due to its efficiency and low cost it represents an alternative to conventional drug sensitivity testing through culture. This test has been successfully used in several locations worldwide with high sensitivity rates for RIF (> 95.5%) and isoniazid (> 81.8%) resistance [8-11] and 100% specificity [8-11]. In recent times, attention has been devoted to this new nucleic acid amplification diagnostic technologies, owing to their rapidity, sensitivity and specificity – the Genexpert MTB/RIF. The Xpert assay uses heminested real-time polymerase chain reaction (PCR) to amplify an M.Tuberculosis – specific sequence of the rpoB gene. To determine rifampicin resistance, the rifampicin resistance – determining region of rpoB gene is probed with molecular beacons [12]. Thus the detection of resistance to rifampicin (the main armament against TB) can be achieved in two hours. The purpose of this study was to determine the prevalence of rifampicin resistance in our setting using the automated system of Genexpert test.

## Methods

The study was done in TB Referral Hospital Igbogene on the outskirts of Yenagoa which is the capital of Bayelsa State Nigeria. The state covers an area of 9,415.8 square kilometres. It has a population of 1,704,515 (2006 census figure). It accounts for 1.2% of the Nigeria’s total population [13]. The hospital serves the state and neighbouring states.

**Specimens**: All sputum specimens that were submitted to the TB referral hospital laboratory from January to December 2016 of patients who were 15 years and above were included in the study.

**XPERT procedure (MTB/RIF assay):** Sample reagent was added in a 2:1 ratio to untreated sputum and in a 3:1 ratio to decontaminate sputum pellets. The additional sample reagent in pellets was necessary to meet the volume requirements for the assay sample. The closed sputum container was manually agitated twice during a 15-minute period at room temperature before 2ml of the inactivated material was transferred to the test cartridge (equivalent to 0.7ml of untreated sputum or 0.5ml of decontaminated pellets). Cartridges were inserted into the test platform which was located in the microscopy room. Test platform (Cepheid, Sunnyvale, CA) is an integrated diagnostic device that performs sample processing and heminested real-time polymerase chain reaction (PCRS) analysis in a single hands-free step for the diagnosis of tuberculosis and rapid detection of RIF resistance in specimens. The electronic results were sent directly from the MTB/RIF test system to the central database and read after 90 minutes.

**Statistical analysis**: This was by sample percentages using Microsoft Excel 2010 for windows.

**Ethical approval**: This study was approved by the Research and Ethics Committee of the State Ministry of Health.

## Results

This study included 446 sputum specimens, all of which were sent to TB Referral Hospital Igbogene, Yenagoa between January to December 2016. Overall, 102 (22.9%) of the sputum specimens were positive for *Mycobacterium tuberculosis* (Table 1); out of which 15 (14.7%) showed rifampicin resistance (Table 2).

**Table 1:** Frequency of *M. tuberculosis* (MTB) detection by GeneXpert

	**Frequency**	**Percentage**
MTB positive	102	22.9
MTB Negative	344	77.1
Total	446	100

**Table 2:** Frequency of *M. tuberculosis* (MTB) resistance to Rifampicin

	**Frequency**	**Percentage**
Sensitive	87	85.3
Resistance	15	14.7
Total	102	100

## Discussion

MDR-TB is TB with bacilli resistant to at least isoniazed and rifampicin, the main anti-tuberculosis drugs. It has become an important concern for TB control in many countries, especially in low-income countries where the burden of other competing diseases like malaria, enteric fever, meningitis etc? is high [8, 14]. With the advent of the Xpert MTB/RIF testing it is now readily possible to rapidly determine TB bacilli susceptibility to common anti TB drugs [8, 15].

Drug resistant TB develops from the inadequate treatment of active pulmonary TB. There are multiple reasons for inadequate therapy; poor prescribing practices with insufficient treatment duration and poor drug selection are well-recognised contributors [1, 8]. Systemic problems, through inadequate public health resources and unpredictable drug supplies also play a role. In addition, irregular medication intake whether from insufficient patient education, adverse events or socio-economic determinants – contribute to resistance [8, 15]. There are also a significant proportion of patients who acquire drug resistant disease because they live in an environment with high prevalence of drug resistance disease. This particular factor makes our finding of high rates of MDR-TB in our samples to be worthy of note. We got a frequency of 14.7% that is, 15 of the 102 specimens that were MTB positive had MDR-TB; at least to isoniazed and rifampicin since the genotypic analysis of rpoB for RIF resistance is thought to be sufficient for evaluating the public health threat of drug resistance TB.

Otu A et al in Calabar, Nigeria reported a drug resistance of 42% in at least one drug in the pulmonary TB patients tested in Calabar, South-South Nigeria. They found mono resistance rate 7% to ethambutol, 7% to streptomycin but no mono resistance to rifampicin by culture [14]. Our study, using the Xpert MTB/RIF assay, tested for resistance to rifampicin only. With Xpert assay, this study obtained rifampicin resistance frequency of 14.7%. The difference in method of testing for rifampicin resistance does not allow for direct comparison. Conventional laboratory techniques like direct microscopy for the diagnosis of tuberculosis are far from being sensitive. Moreover, cultures are time-consuming, require biosafety measures, and need trained laboratory personnel. We have in our study employed the molecular techniques which, we know, have greatly changed the field of tuberculosis diagnosis and have proven to yield rapid results while being highly sensitive [3]. The Genexpert assay used in our study targets the rifampicin resistance associated rpoB gene, region by heminested PCR with three specific Primers and combines the sensitive detection of M.tuberculosis DNA and determination of RMP resistance. In same vein, the hands on time is short due to automation of bacterial lysis, DNA extraction, real time PCR amplification and amplicon detection in a single system. Boehme and others showed the high sensitivity of over 97% and specificity of the Xpert assay for pulmonary specimens [3].

Otu A et al in their study had attributed the low frequency of resistance to rifampicin to possibly be due to the new history of use of rifampicin in African countries [14]. The narrative may have changed many years on. There has always been a need in the global control of tuberculosis to intensify the development of rapid diagnostic test for MDR-TB. This work further strengthens this need as MDR-TB can be rapidly identified and treatment appropriately instituted. One limitation of this study is the fact that it was limited to the TB Referral Hospital in Igbogene Yenagoa and its catchment area. A larger nationwide survey using the Xpert assay will provide a better estimate of MDR-TB in Nigeria.

## Conclusion

There is a progressive increase in the frequency of MDR-TB especially to rifampicin in our setting. There is need to replicate this laboratory facility for rapid detection of MDR-TB in many more zones of the country for effective coverage.

### What is known about this topic

- Nigeria is a high burden country for MDR- TB which poses a major challenge to the control of TB;
- Drug resistant TB leads to prolonged duration of the disease and with associated increased mortality;
- The diagnosis of MDR-TB in our resource poor setting had mainly been by culture method which is both cumbersome and costly.

### What this study adds

- The prevalence of MDR-TB in our setting using GeneXpert instead of the commonly used culture method;
- The prevalence of MDR-TB in our setting is higher than the predicted value for the country by WHO.

## Competing interests

The authors declare no competing interests.

## References

[1] World Health Organization (2007). The global MDR-TB & XDR-TB response plan 2007-2008.

[2] World Health Organization (2010). Treatment of tuberculosis: guidelines..

[3] Boehme CC, Nabeta P, Hillemann D, Nicol MP, Shenai S, Krapp F (2010). Rapid molecular detection of tuberculosis and rifampin resistance. N Engl J Med..

[4] Chan ED, Iseman MD (2008). Multidrug-resistant and extensively drug-resistant tuberculosis: a review. Curr Opin Infect Dis..

[5] Helb D, Jones M, Story E, Boehme C, Wallace E, Ho K (2010). Rapid detection of Mycobacterium tuberculosis and rifampin resistance by use of on-demand, near-patient technology. J Clin Microbiol..

[6] Maxmen A (2010). Fighting the monster. Nature..

[7] World Health Organisation (2017). Nigeria: Tuberculosis Country profile 2016..

[8] Anek-vorapong R, Sinthuwattanawibool C, Podewils LJ, McCarthy K, Ngamlert K, Promsarin B (2010). Validation of the GenoType^®^ MTBDR plus assay for detection of MDR-TB in a public health laboratory in Thailand. BMC Infect Dis..

[9] Causse M, Ruiz P, Gutierrez J, Zerolo J, Casal M (2008). Evaluation of new GenoType^®^ MTBDRplus for detection of resistance in cultures and direct specimens of Mycobacterium tuberculosis. Int J Tuberc Lung Dis..

[10] Huang W-L, Chen H-Y, Kuo Y-M, Jou R (2009). Performance assessment of the GenoType MTBDRplus test and DNA sequencing in detection of multidrug-resistant Mycobacterium tuberculosis. J Clin Microbiol..

[11] Lacoma A, Garcia-Sierra N, Prat C, Ruiz-Manzano J, Haba L, Roses S (2008). GenoType MTBDRplus assay for molecular detection of rifampin and isoniazid resistance in Mycobacterium tuberculosis strains and clinical samples. J Clin Microbiol..

[12] El-Hajj HH, Marras SA, Tyagi S, Kramer FR, Alland D (2001). Detection of rifampin resistance in Mycobacterium tuberculosis in a single tube with molecular beacons. J Clin Microbiol..

[13] City populations worldwide Bayelsa Population 2017..

[14] Otu A, Umoh V, Habib A, Ameh S, Lawson L, Ansa V (2013). Drug resistance among pulmonary tuberculosis patients in Calabar, Nigeria. Pulm Med..

[15] Onyedum CC, Alobu I, Ukwaja KN (2017). Prevalence of drug-resistant tuberculosis in Nigeria: a systematic review and meta-analysis. PLoS One..

